# A High-Fat and High-Fructose Diet Exacerbates Liver Dysfunction by Regulating Sirtuins in a Murine Model

**DOI:** 10.3390/life14060729

**Published:** 2024-06-05

**Authors:** Zehuan Ding, Jian Zhang, Mahua Choudhury

**Affiliations:** Department of Pharmaceutical Sciences, Irma Lerma Rangel School of Pharmacy, Texas A&M University, College Station, TX 77843-1114, USA

**Keywords:** high-fat high-fructose diet, sirtuin, metabolic dysfunction-associated steatotic liver disease, obesity, liver fibrosis

## Abstract

Metabolic dysfunction-associated steatotic liver disease (MASLD) is rapidly emerging as the most prevalent chronic liver disease, closely linked to the escalating rates of diabesity. The Western diet’s abundance of fat and fructose significantly contributes to MASLD, disrupting hepatic glucose metabolism. We previously demonstrated that a high-fat and high-fructose diet (HFHFD) led to increased body and liver weight compared to the low-fat diet (LFD) group, accompanied by glucose intolerance and liver abnormalities, indicating an intermediate state between fatty liver and liver fibrosis in the HFHFD group. Sirtuins are crucial epigenetic regulators associated with energy homeostasis and play a pivotal role in these hepatic dysregulations. Our investigation revealed that HFHFD significantly decreased Sirt1 and Sirt7 gene and protein expression levels, while other sirtuins remained unchanged. Additionally, glucose 6-phosphatase (G6Pase) gene expression was reduced in the HFHFD group, suggesting a potential pathway contributing to fibrosis progression. Chromatin immunoprecipitation analysis demonstrated a significant increase in histone H3 lysine 18 acetylation within the G6Pase promoter in HFHFD livers, potentially inhibiting G6Pase transcription. In summary, HFHFD may inhibit liver gluconeogenesis, potentially promoting liver fibrosis by regulating Sirt7 expression. This study offers an epigenetic perspective on the detrimental impact of fructose on MASLD progression.

## 1. Introduction

The significant and escalating health challenges posed by dietary habits in the Western world underscore the need for a comprehensive understanding of the intricate interplay between nutrition and health outcomes. One prominent aspect of this dietary landscape is the considerable intake of added sugars, which constitute approximately 15% of the total energy intake in the average Western diet [1]. This prevalence of added sugars is a critical factor contributing to the broader health crisis. As research indicates, diets rich in both fats and high fructose content are particularly implicated in the rising incidence of obesity [1]. The rise in obesity rates and the adoption of a Western diet have been identified as pivotal factors in the increasing prevalence of metabolic dysfunction-associated steatotic liver disease (MASLD), formerly known as nonalcoholic fatty liver disease (NAFLD). In the United States, MASLD is the most common chronic liver disease and is estimated to affect approximately 30% of the population [1]. The intricate relationship between dietary patterns and liver health is illuminated by the progression of MASLD, which unfolds as a complicated journey from metabolic dysfunction-associated fatty liver, characterized by hepatic steatosis, to more severe conditions. The initial stage, metabolic dysfunction-associated fatty liver, is characterized by the presence of hepatic steatosis. Additional factors, including insulin resistance and oxidative stress, can lead to metabolic dysfunction-associated steatohepatitis (MASH); this is characterized by hepatic inflammation, cell damage, and can ultimately progress to liver fibrosis as well as cirrhosis [2]. Nearly 20% of those with MASLD go on to develop MASH; additionally, more than 20% of those with MASH may develop liver cirrhosis [3]. MASLD is a multifactorial disease that results from the interplay of environmental and social factors [4]. However, the underlying pathophysiological processes linked with epigenetic regulations remain unclear. More research is imperative to prevent the progression of these liver dysregulations.

The high-fat diet (HFD) is a widely used model to study obesity and MASLD in rodents; however, the nutrient composition of the HFD is not sufficient to match the Western diet. HFD exposure alone often fails to replicate the MASH phenotype [5]. To address this, the role of carbohydrates, especially fructose, is gradually gaining recognition as being a determining factor in the development of MASLD and other metabolic diseases [6]. Fructose is a highly lipogenic sugar since its metabolism bypasses the rate-limiting step of glycolysis, and therefore activates de novo lipogenesis [7]. Fructose is also strongly associated with the progression and severity of MASLD [8,9]. Evidence has emerged exhibiting that fructose can also elicit hepatic oxidative stress and systemic insulin resistance [10,11,12]. It has been demonstrated that high sugar content in the form of fructose and sucrose combined with high fat can induce liver steatosis, inflammation and further worsen fibrosis [10,13,14]. A recent study suggests that fructose feeding promotes mild fibrosis with steatosis and portal inflammation of hepatocytes in rabbit livers [15]. In addition, our recently published study demonstrated that the chronic high-fat high-fructose diets (HFHFDs), when administered for 20 weeks, led to the surpassing of the hepatic steatosis stage, potentially to an intermediate point between fatty liver and fibrosis [16]. However, the molecular mechanism is still yet to be investigated.

Epigenetic regulation affects gene accessibility and expression through histone and protein modification, such as acetylation or deacetylation of terminal lysine residues. Sirtuins are a group of class III histone deacetylases that play a critical role in cellular metabolism. There are seven mammalian homologs of sirtuins (Sirt1–7), each with distinct subcellular regions, cellular functions, and enzymatic activations [17,18]. Sirtuins function as nutrient sensors and regulators of glucose and lipid metabolism, making them vital mediators of liver metabolism [19,20,21,22]. Research has shown that Sirt1 plays a significant role in liver metabolism [23]. Hepatic Sirt1 knockout has been found to exacerbate steatosis and inflammation in the liver. Sirt1 has also been found to promote apoptosis of activated stellate cells and block hepatic stellate cell activation [24,25], leading to the amelioration of liver fibrosis. Additionally, Sirt1 is involved in inhibiting adipogenesis in adipose tissue and activating gluconeogenesis in the liver [26]. We have previously shown that Sirt3 activity decreases in mice fed a chronic high-fat diet, leading to impaired mitochondrial function and hyperacetylation of proteins in the liver [27]. Mice lacking Sirt3 developed accelerated obesity, insulin resistance, and steatohepatitis compared to wild-type mice when fed a chronic high-fat diet [28]. On the other hand, Sirt4 and Sirt5 have been found to suppress fatty acid oxidation [29,30,31]. However, Sirt7’s role in the liver remains controversial. A study using Sirt7 knockout model demonstrated that Sirt7 suppresses ER stress and reverts the fatty liver disease in diet-induced obese mice [32]. Conversely, a different knockout model has shown that Sirt7 promotes hepatic fatty acid uptake and triglyceride synthesis [33]. The above strongly suggests that sirtuins play a crucial role in regulating the progression of liver aberrations in multiple aspects. In this study, we investigated sirtuin regulation in an HFHFD model to examine the epigenetic role of sirtuins in MASLD pathogenesis. Our results provide novel insights into MASLD pathogenesis and highlight the role of sirtuins as a valuable therapeutic target for MASLD.

## 2. Materials and Methods

### 2.1. Mice

C57Bl/6 male mice (5 weeks old) were purchased from the Jackson Laboratory. Mice were fed an ad libitum chow diet and water (Cat#PicoLab Rodent Diet 5053, Lab Supply, Fort Worth, TX, USA), and acclimated in a 12:12 h light/dark cycle temperature-controlled room for one week. Mice were then switched to a low-fat diet (LFD) (10% energy from fat, Research Diet D12450H), HFD (45% energy from fat, Research Diet D12451Y), or HFHFD (45% energy from fat, 17% energy from fructose, Research Diet D15041701) and maintained for 20 weeks (10 mice per group) [34]. At the end of the 20-week diet regimen, mice were euthanized under anesthesia after 6 h of fasting. The liver tissues were excised, snap-frozen in liquid nitrogen, and stored at −80 °C. The mice and tissue used in this study are the same as in our previous study [16]. All procedures were approved by Texas A&M Institutional Animal Care and Use Committee.

### 2.2. Real-Time Quantitative PCR

RNA was extracted from liver tissues according to the Qiagen miRNeasy Mini Kit (Cat#217004, Qiagen, Germantown, MD, USA) protocol as per the manufacturer’s instructions. A total of 1 μg of mRNA was reverse transcribed using the High-Capacity cDNA Reverse Transcription Kit (Cat# 4368814, Life Technologies, Carlsbad, CA, USA). Quantitative real-time PCR was used to determine the expression levels of gene transcripts using SYBR Green PCR Master Mix (Cat# A25742, Life Technologies, Carlsbad, CA, USA). Relative gene expression was normalized to *β*-*actin* and represented as a fold change [35]. Each experiment was repeated once to confirm the result. The primer sequences used for PCR are listed in Table S1.

### 2.3. Western Blot

Liver tissues were homogenized in an RIPA lysis buffer (50 mM Tris, pH 8, 150 mM NaCl, 1% Nonidet P-40, 0.1% SDS, 0.5% sodium deoxycholate), 0.5 mM PMSF, and 1X protease and phosphatase inhibitor (10 µL/mL). After centrifugation at 15,000× *g* for 15 min at 4 °C, the clear supernatants were transferred to new tubes and stored at −80 °C. The protein content of the clarified lysate was determined using Pierce™ BCA Protein Assay Kits (Cat#23225) from Thermo Fisher Scientific (Waltham, MA, USA). Isolated proteins were denatured in an SDS gel buffer, separated by SDS-PAGE, and immunoblotted for respective targets. Goat anti-rabbit or anti-mouse IRDye 680 (Cat#926-32210) or IRDye 800 (Cat#926-32211) secondary antibodies from LiCor were used for the detection and quantification of immunoblots. Membranes were imaged using a LiCor Odyssey scanner, and blots were analyzed by Image Studio 4.0 analytical software (LiCor, Lincoln, NE, USA), as previously described [36,37]. Each experiment was repeated once to confirm the result. Primary antibodies included Sirt1 (Cat#2028), Sirt2 (Cat#D4S6J), Sirt3 (Cat#5490), Sirt5 (Cat#8779), Sirt6 (Cat#12486), Sirt7 (Cat#5360), and α-Tubulin (Cat#3873) from Cell Signaling Technologies (Danvers, MA, USA), Sirt4 (Cat#PA5-96727) and β-actin (Cat#PA5-59497) from Invitrogen (Carlsbad, CA, USA), and glucose 6-phosphatase (G6Pase) (Cat#A20193) from Abclonal (Woburn, MA, USA).

### 2.4. Chromatin Immunoprecipitation (ChIP)

ChIP was performed using EZ-Magna ChIP^TM^ HiSens Kit (cat#17-10461, Millipore, Burlington, MA, USA), according to the manufacturer’s protocol. Anti-Acetylated Histone H3 Lysine 18 (acH3K18) antibody (Cat#ab1191) from Abcam (Waltham, MA, USA) was used for ChIP. Briefly, tissues were dissected and underwent crosslinking with 1% formaldehyde for 10 min at room temperature, followed by quenching with 125 mM glycine for 5 min. After two cold PBS washes, the fixed tissues were homogenized and then resuspended in a sonication buffer containing protease inhibitors. The tissue suspension was sonicated for fragmentation. For immunoprecipitation, the diluted chromatin was incubated with Anti-acH3K18 antibody or normal rabbit IgG immobilized on Protein A/G Sepharose beads for 12 h at 4 °C with constant rotation. Beads were washed with wash buffer and mixed with ChIP Elution Buffer and protease K, then added to the samples and incubated at 65 °C for 2 h and then at 95 °C for 15 min. Samples were centrifuged for 1 min at 4 °C, the supernatants were transferred to new tubes, and then they were subjected to quantitative PCR using a mouse G6Pase promoter and an intergenic control. Each experiment was repeated once to confirm the result. The primer sequences are listed in Table S1. All ChIP signals were calculated as fold enrichment by the 2^−∆∆Ct^ method and normalized to the intergenic control.

### 2.5. Statistical Analysis

All data are presented as the mean ± standard error. Comparisons between the groups were performed using one-way ANOVA unless otherwise specified, followed by Tukey’s test using Prism Graphpad (version 7.0). *p*-values less than 0.05 were considered statistically significant in all experiments.

## 3. Results

### 3.1. Hepatic Sirt1 and Sirt7 Gene Expression Is Downregulated upon HFHFD Feeding

In our previous article, we evaluated lipid metabolism and liver fibrosis markers. We found a decrease in fatty acid synthesis, but we did not observe any changes in fibrosis [16]. To confirm the impact of HFHFD on liver metabolism, we measured several other markers related to lipid metabolism, liver inflammation, and fibrosis. Similarly, we observed significant downregulation of Acetyl-CoA carboxylase (*Acc*) in both HFD and HFHFD groups, but Matrix Metallopeptidase 2 (*Mmp2*), which promotes liver fibrosis, was not changed (Supplemental Figure S1). We also observed that the gene levels of Tumor Necrosis Factor Alpha (*Tnfα*) were increased in the HFHFD group (Supplemental Figure S1), suggesting an HFHFD-induced inflammatory condition in the liver, which served as an essential trigger for liver fibrosis.

**Figure 1:**
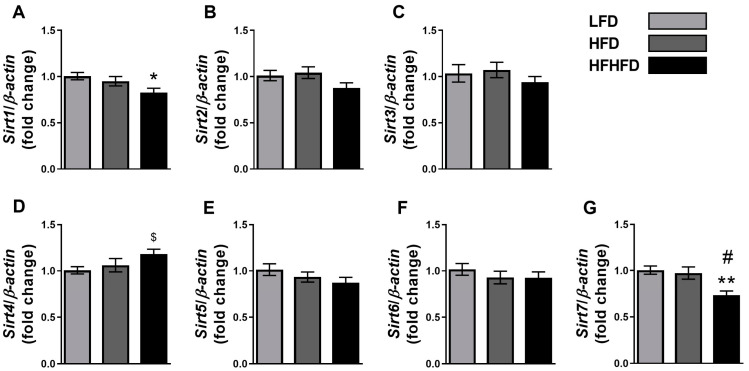
HFHFD differentially altered sirtuins gene expressions in the liver. Fold change of gene expression of (**A**) *Sirt1*, (**B**) *Sirt2*, (**C**) *Sirt3*, (**D**) *Sirt4*, (**E**) *Sirt5*, (**F**) *Sirt6*, and (**G**) *Sirt7* in livers of LFD-, HFD-, and HFHFD-fed mice. Gene expression was normalized to *β*-*actin*. *n* = 7–8, * *p <* 0.05, ** *p <* 0.01 compared to LFD group. ^#^ *p* < 0.05 compared to HFD group. ^$^ *p* < 0.05 with Student’s *t*-test compared to LFD group.

Sirtuins play a critical role in regulating genes that are involved in energy metabolism in the liver [38]. Dysregulation of several sirtuin proteins is associated with MASLD progression [17,33]. To evaluate the impact of high-fat or high-fructose feeding on liver metabolism, we evaluated the mRNA expression changes in sirtuins in the livers of LFD-, HFD-, or HFHFD-fed mice. Our findings revealed a significant decrease in hepatic gene expression of *Sirt1* in the HFHFD group compared to the LFD group (Figure 1A). However, there were no differences between HFD and HFHFD. Conversely, *Sirt7* exhibited a notable downregulation of transcript levels in the HFHFD group compared to both the LFD (*p <* 0.01) and HFD groups (*p* < 0.05, Figure 1G). We also observed an increased trend of *Sirt4* in the HFHFD group compared to the LFD group (*p* = 0.016 with Student’s *t*-test). No other sirtuins displayed altered gene expression in HFHFD when compared to LFD (Figure 1B–F). Interestingly, none of the sirtuins were altered in the HFD group. These results suggest that *Sirt1* and *Sirt7* are the major sirtuins that are involved in the liver under the influence of HFHFD.

### 3.2. Hepatic Sirt1 and Sirt7 Protein Expression Is Downregulated upon HFHFD Feeding

**Figure 2:**
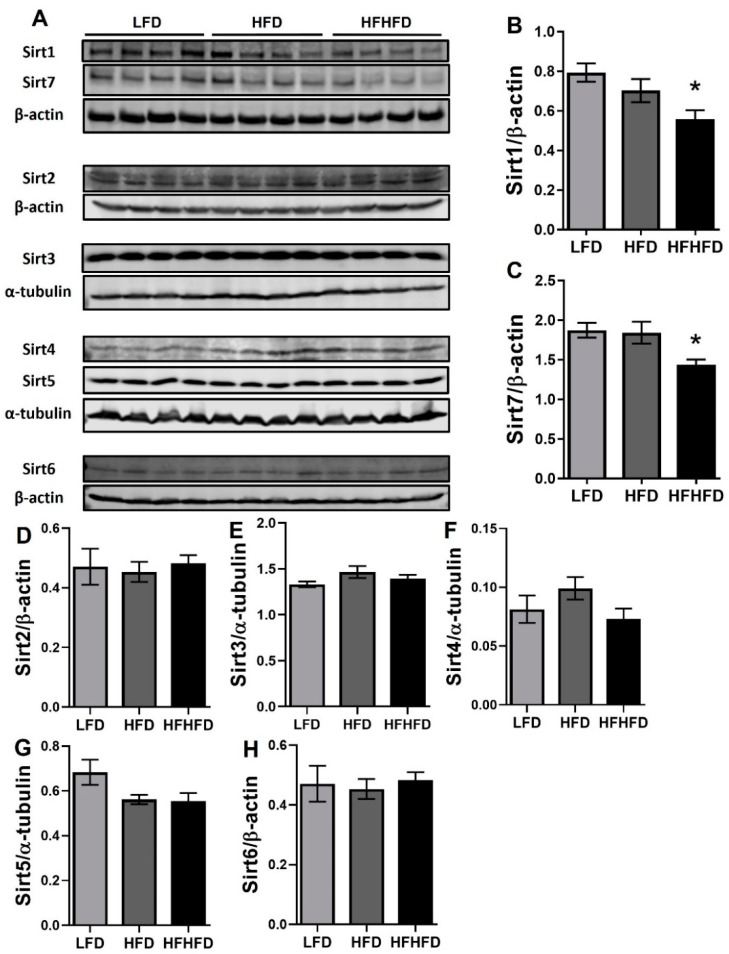
Decreased Sirt1 and Sirt7 protein expressions in HFHFD-fed mice liver. (**A**) Representative protein expression of Sirt1-7, β–actin, and α-tubulin in livers of LFD, HFD, and HFHFD mice. (**B**,**C**) Densitometry analysis of (**B**) Sirt1 and (**C**) Sirt7 protein expression. Expression was normalized to β-actin. (**D**–**H**) Densitometry analysis of (**D**) Sirt2, (**E**) Sirt3, (**F**) Sirt4, (**G**) Sirt5, and (**H**) Sirt6 protein expression. Expression was normalized to β-actin or α-tubulin. *n* = 7–8, * *p <* 0.05 compared to LFD group.

To validate the expression changes detected at the transcriptional level, we further measured the expression of sirtuins at the protein level. Consistent with the gene expression data, protein levels of Sirt1 (*p* < 0.05, Figure 2A,B) and Sirt7 (*p* < 0.05, Figure 2A,C) were significantly lower in the HFHFD group compared to the LFD group, but no difference was observed in the HFD group when compared to LFD or HFHFD. The protein expressions of other sirtuins were not observed to be altered in either HFD or HFHFD when compared to the LFD group. Downregulation of Sirt1 and Sirt7 indicates that the HFHFD may disrupt the hepatic metabolism and contribute to liver deregulation progression through epigenetic modification.

### 3.3. G6Pase Promoter Hyperacetylation Suppresses Its Expression upon HFHFD Feeding

**Figure 3:**
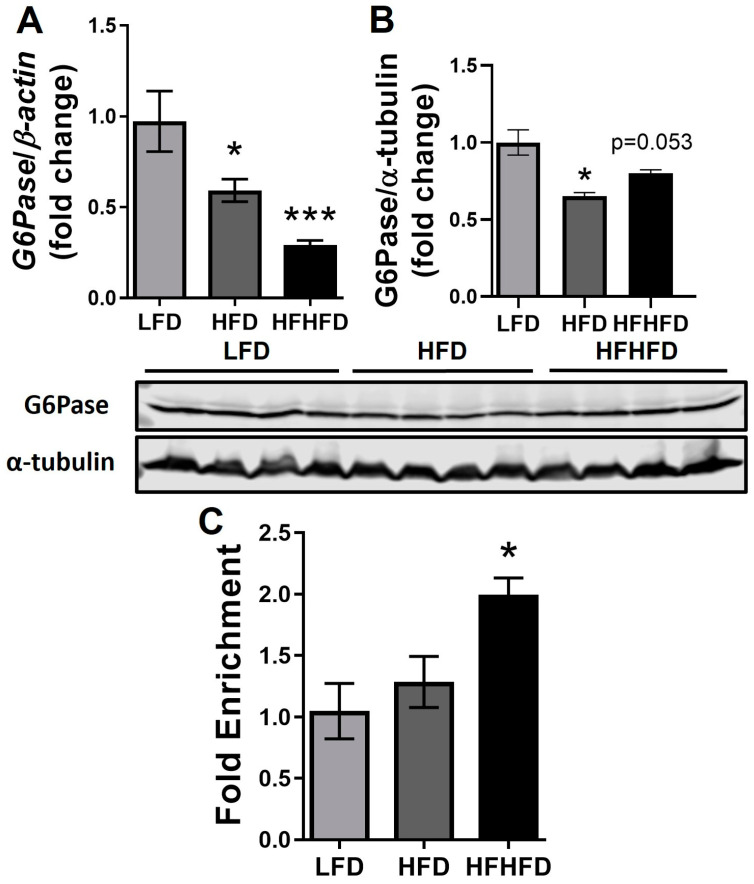
Gluconeogenic maker was inhibited through promoter histone hyperacetylation in the HFD-HF mice liver. (**A**) Gene expressions of *G6Pase* in livers of LFD, HFD, and HFD-HF groups. Expression was normalized to *β*-*actin*. (**B**) Protein expression and densitometry of G6Pase in livers of LFD, HFD, and HFD-HF mice. Expression was normalized to α-tubulin. (**C**) Fold enrichment of G6Pase promoter in H3K18Ac ChIP assay. *n* = 8 for (**A**,**B**); *n* = 3 for (**C**); one-way ANOVA with Tukey’s post hoc test; * *p <* 0.05, *** *p <* 0.001 compared to LFD.

G6Pase is a key regulator of hepatic gluconeogenesis [39]. Here, we examined the expression of G6Pase to evaluate the effect of HFHFD. Interestingly, HFHFD significantly suppressed the gene expression of *G6Pase* in the liver compared to LFD (*p* < 0.001, Figure 3A). The HFD group also exhibited a decrease in *G6Pase* expression compared to the LFD group (*p* < 0.05, Figure 3A), but the fold change was less remarkable compared to the trend observed in the HFHFD group. To further validate the result, we measured the protein level of G6Pase. A significantly decreased level of G6Pase was observed in the HFD group compared with the LFD group, and the HFHFD group showed a decreasing trend (*p* = 0.053) (Figure 3B), suggesting that the downregulation is consistent at gene and protein levels. Sirt7 is an NAD^+^-dependent deacetylase whose substrates include acH3K18 [40]. It has been demonstrated that Sirt7 promotes G6Pase expression via H3K18 deacetylation at its promoter region [41]. To confirm the possible regulation of G6Pase through deacetylation by Sirt7, we probed this interaction using a ChIP assay. We demonstrated that the livers from the HFHFD-fed group exhibited significantly increased acetylation of H3K18 in the G6Pase promoter compared to the LFD (*p <* 0.05, Figure 3C) group. No changes were found between LFD and HFD. These data indicate that HFHFD inhibits G6Pase through sirtuin-regulated histone modification.

## 4. Discussion

MASLD has become a global health burden. It is the most prevalent type of chronic liver disease in Western countries [42]. The MASLD pandemic is tightly associated with traditional Western diets rich in saturated fat and fructose, both of which have been linked to the development of severe liver diseases [43]. While the prevalence of MASLD is well established, the underlying processes propelling its development remain elusive. This knowledge gap highlights the imperative focus for further research to unravel the intricacies of MASLD pathogenesis. Understanding the epigenetic dysregulation induced by environmental triggers, especially lifestyle factors, and if there are any new pathways other than the genetics that contribute to MASLD will not only enhance our comprehension of the disease but also pave the way for targeted interventions to impede its progression. There is a growing recognition of the role of epigenetic mechanisms where the environment plays a role, including histone modifications in MASLD [44,45]. As mentioned earlier, fructose undergoes a distinct metabolic pathway in the liver, bypassing the rate-limiting step of glycolysis and promoting de novo lipogenesis. The correlation between fructose consumption and the onset of MASLD carries significant implications for public health. Our prior research demonstrated that introducing fructose into the diet exacerbates liver metabolism issues, as evidenced by increased body weight and worsened insulin sensitivity [46]. In this current study, we elucidate the impact of the fructose component in an HFHFD on the progression of liver disease through sirtuin regulation.

**Figure 4:**
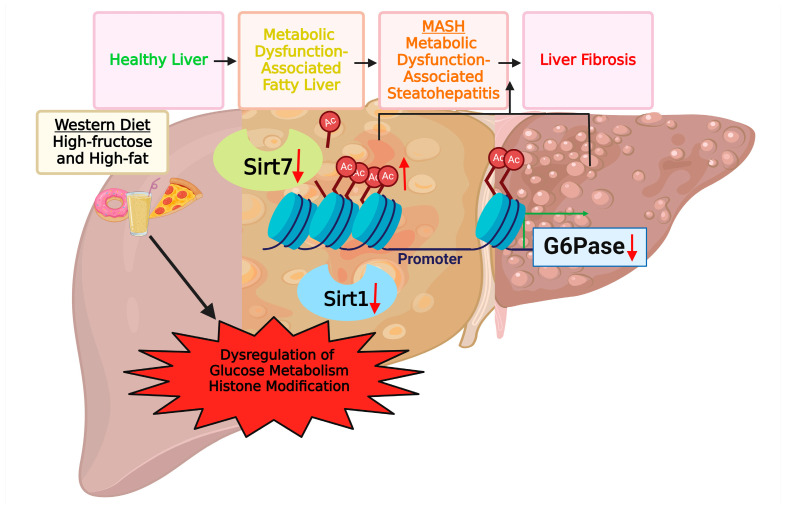
Proposed model of Western diet-induced liver dysfunction via epigenetic regulation. The proposed model posits that a Western diet characterized by high fructose and fat content exerts inhibitory effects on the expression of Sirt1 and Sirt7. This inhibition, in turn, reduces their deacetylation activity during histone modification. This dysregulation has a significant impact on hepatic pathogenetic progression, transitioning from the early stage of metabolic dysfunction-associated fatty liver to the more advanced state of steatohepatitis, culminating in the induction of liver fibrosis. Importantly, this series of events involves dysregulation of histone modification, specifically inhibiting G6Pase expression via promoter hyperacetylation. This inhibition of Sirt1 and Sirt7 has far-reaching implications for glucose metabolism, indicating an intermediate status that lies between steatohepatitis and liver fibrosis. The intricate interplay between dietary components, epigenetic regulation, and the consequential impact on histone modification sheds light on the potential molecular mechanisms driving the progression of liver dysfunction induced by a Western diet. Created with BioRender.com (accessed on 1 June 2024).

To elucidate the regulation of sirtuins under the context of HFHFD-induced MASLD, our study has demonstrated the predominant influence of Sirt1 and Sirt7 expression, with an upsurge in Sirt4 only at the transcriptional level (Figure 1 and Figure 2). Sirtuins belong to the class III histone deacetylases, depending on NAD+ [47]. They are integral players in cellular metabolism and energy homeostasis regulation [48,49,50]. Even though Sirt4 or other sirtuins did show roles in the liver metabolism [51,52], the lack of significant changes of these sirtuins here underscored the predominant influence of Sirt1 and Sirt7 under HFHFD, suggesting a selective influence on sirtuin expression under specific diet composition. Many studies have shown decreased expression of Sirt1 in fatty liver [53,54]. The roles of Sirt1 in fatty liver disease include the regulation of lipogenesis, fatty acid β-oxidation, oxidative stress, and hepatic inflammation [17]. Recently, Sirt1 has also been shown to play an important role in hepatic fibrosis [24,25,55]. Consistent with previous studies, both protein and gene expression of hepatic Sirt1 were downregulated in HFHFD mice. On the contrary, the controversy surrounding Sirt7’s role in hepatic dysfunction adds complexity. Studies on mouse liver lacking exons 4-9 in Sirt7 exhibited resistance to HFD-induced liver steatosis [33], while those with exons 4-11 replaced by the LacZ gene demonstrated elevated steatosis via regulation of the ubiquitin-proteasome pathway [32]. Although Sirt7’s role in hepatic fibrosis remains elusive, its downregulation in normal human lung fibroblasts [56] and its regulatory role in lung fibroblast fibrotic phenotype [57] hint at potential implications in fibrosis development. Intriguingly, in high-fructose-fed prediabetic rats, both Sirt1 mRNA and protein levels, along with Sirt7 mRNA levels, were severely downregulated [58]. Our study, combined with prior findings, suggests that HFHFD induces hepatic dysfunction by inhibiting Sirt1 and Sirt7 expression, propelling the liver into an intermediate stage conducive to fibrosis development (Figure 4).

Additionally, the nuclear localization of Sirt1 and Sirt7 underscores their potential epigenetic function, given their involvement in histone acetylation regulation [59]. For example, a study shows that Sirt1 modulates the acetylation patterns of histones H3 and H4 in breast cancer [60]. Furthermore, although other sirtuins’ expression levels remain unaffected by HFHFD feeding in our study, it is noteworthy to explore their potential roles in liver disease under different diet conditions, considering their established importance in liver dysregulations [61,62]. For instance, Sirt3 has been implicated in mitigating liver damage induced by a high-fat diet, highlighting sirtuins’ broader impact beyond Sirt1 and Sirt7 in maintaining hepatic homeostasis [63]. Another important consideration in this scenario is that post-translational modifications among sirtuins, such as phosphorylation and acetylation, may influence their enzymatic activity without requiring changes in expression levels. We previously showed that HFD decreased Sirt3 activity but not protein expression in fatty liver [27]. For another example, Sirt6 undergoes phosphorylation at Tyr294 and Ser303, which may influence its function, particularly in DNA repair and gene regulation [64]. Similarly, Sirt2 can be acetylated by the acetyltransferase p300, leading to the inhibition of its catalytic activity [65]. In future studies, understanding the nuanced interplay among not only Sirt1 and Sirt7 but also other sirtuins may offer a more comprehensive understanding of their collective impact on liver health and MASLD progression.

Furthermore, our investigation revealed that there were no significant alterations observed in any of the sirtuins in the HFD group, which contrasts with findings from previous studies. For example, Deng et al. have demonstrated that Sirt1 expression is significantly reduced in liver induced by a 3-month high-fat diet (50% of energy derived from fat) feeding in rats [66]. This discrepancy in diet composition or treatment duration may contribute to varying results. Another reason is that our comparison is based on LFD, which has a higher fat content compared with a regular chow diet. Healthy humans do consume some amount of fat in their day-to-day lives. Furthermore, the use of an LFD for diabetes-obesity research is also strongly encouraged instead of standard chow diets. This aligns with the National Institutes of Health’s mission of promoting research rigor and reproducibility [67]. As a result, we decided to use LFD as our baseline comparison to simulate a realistic human diet. While many studies have utilized high-fat diets to study MASLD, some have reported failures to generate steatohepatitis or fibrosis phenotypes [68,69]. Incorporating additional components, such as fructose or sucrose, which mimic aspects of the Western diet, has yielded success [70]. However, long-term investigations into the effects of HFHFD on liver function are limited. Furthermore, the intricate composition of the diet may obscure the true impact of fructose ingestion. Our study sheds light on the detrimental effects of combining high fat and high fructose, offering insights into the role of fructose in exacerbating MASLD phenotypes compared to high-fat diets alone.

To evaluate the impact of HFHFD feeding on liver metabolic function, our study revealed a significantly decreased G6Pase expression in the livers of HFHFD male mice (Figure 3), which can subsequentially disrupt glucose homeostasis and exacerbate liver dysfunction. It is well established that G6Pase is one of the rate-limiting enzymes that plays a crucial role in gluconeogenesis [39]. It is predominantly expressed in the liver and kidneys and is tightly regulated to ensure glucose homeostasis [71]. While G6Pase is primarily associated with gluconeogenesis, its involvement in liver inflammation and fibrosis has garnered increasing attention [72]. Hepatic gluconeogenesis is usually thought to be increased in the diet-induced MASLD model [73,74,75]. However, multiple studies have also demonstrated that G6Pase expression is decreased in fibrotic livers in both human and mouse models [39,76,77]. Recent studies have indicated that G6Pase enzymatic activity is decreased in liver cirrhosis [77]. These data combined with our observations display that HFHFD feeding drives the liver to a more severe state, which exhibits characteristics of fibrosis. Interestingly, Sirt7 has already been shown to regulate G6Pase through histone modification at the promoter region [26,40,41]. To further delineate the mechanism of sirtuin-related epigenetic regulation, particularly histone modification, our result demonstrated the increased H3K18 acetylation at the promoter region of G6pase. This alteration potentially contributes to its inhibition. Consistent with our findings, fructose intake has been implicated in the modulation of histone acetylation patterns and sirtuin activity [78]. In fructose-fed diabetic rats, resveratrol, a natural polyphenol, increased Sirt1 expression and deacetylated histone H3 lysine 9, attenuating cardiac hypertrophy and oxidative stress by downregulating NADPH oxidase [79]. Additionally, increased H3K18 acetylation has been associated with the promotion of fibroblast growth factor 21 (FGF21) production [80]. Fructose intake has been shown to dramatically stimulate circulating FGF21 levels [81]. Taken together, these results support a potential association between fructose ingestion and H3K18 acetylation regulation through sirtuins (Figure 4). Taken together with our prior studies implicating the HFHFD-induced modulation of the PPAR-γ pathway via miR-27b-5p, our research highlights the impact of HFHFD on liver metabolism function through different aspects, including transcriptional and epigenetic regulation. While evidence has shown the regulation of sirtuins and PPAR-γ [17,82], further elucidating their relative contributions and potential interplay is crucial for understanding the complex pathogenesis of MASLD.

Prospectively, our findings pave the way for future studies that can expand on our current understanding of sirtuin involvement in diet-induced hepatic dysfunction. One promising avenue is the exploration of targeted interventions aimed at modulating sirtuin activity. Investigating pharmacological or dietary strategies to enhance the expression or activity of protective sirtuins, particularly Sirt1 and Sirt7, could hold therapeutic potential for preventing or mitigating the progression of MASLD [83]. Additionally, considering the potential epigenetic functions of Sirt1 and Sirt7, exploring small molecules or interventions that specifically target histone modifications may open new avenues for precision medicine in liver disease [84]. The complex interplay of sirtuins, specifically Sirt1 and Sirt7, in diet-induced hepatic dysfunction offers a multifaceted landscape for exploration, enriching our understanding of sirtuin dynamics in the context of MASLD. As we continue to unravel the intricacies of sirtuin’s involvement in liver health, the prospect of developing targeted interventions holds promise for addressing the growing global burden of diet-associated liver diseases. Our study further demonstrated that a Western diet model with HFHFD induces liver dysfunction through disturbing sirtuin-mediated pathways, providing novel insights into epigenetic mechanisms potentially involved in an intermediate state toward liver fibrosis. Future research is needed to better understand the specific roles of Sirt7 in the pathogenesis of liver fibrosis progression, which can guide the development of more precise and effective anti-MASLD treatments.

## Data Availability

The original contributions presented in the study are included in the article/Supplementary Material, further inquiries can be directed to the corresponding author.

## Supplementary Materials

The following supporting information can be downloaded at: https://www.mdpi.com/article/10.3390/life14060729/s1, Figure S1: mRNA expression of lipid metabolism and liver fibrosis markers in male liver among the LFD, HFD, and HFD-HF groups for 20 weeks; Table S1: List of primer sequences.
