# Supplementary material for: A High-Fat and High-Fructose Diet Exacerbates Liver Dysfunction by Regulating Sirtuins in a Murine Model
Source: Life (Basel). 2024 Jun 5;14(6):729. doi: 10.3390/life14060729 (PMC11205069; doi:10.3390/life14060729)
Supplement: Supplementary file 1 [file life-14-00729-s001.zip › Figure S1.pdf]

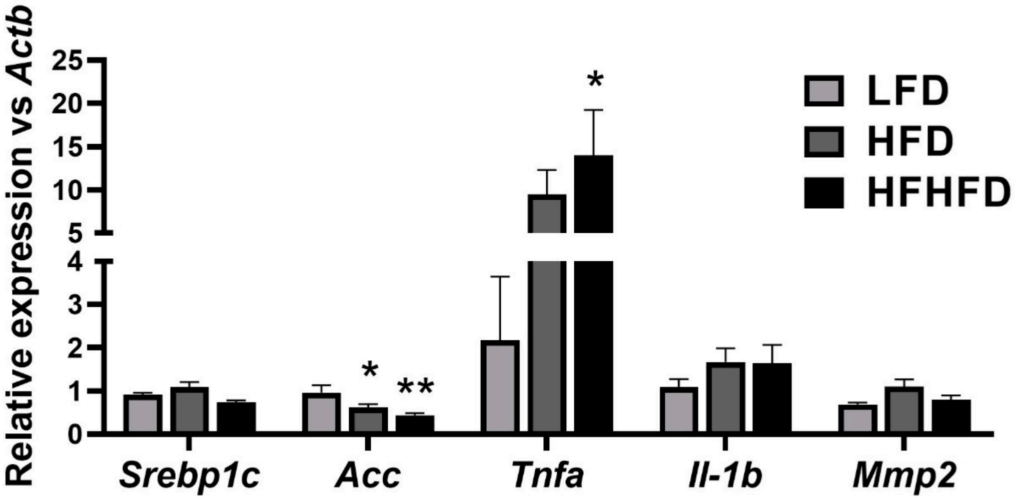

Figure S1. mRNA expression of lipid metabolism and liver fibrosis markers in male liver among the LFD, HFD, and HFD-HF groups for 20 weeks. n = 7 mice/group. One-way ANOVA with Tukey's post hoc test, *p<0.05, **p<0.01 compared to LFD.
