# Supplementary material for: A High-Fat and High-Fructose Diet Exacerbates Liver Dysfunction by Regulating Sirtuins in a Murine Model
Source: Life (Basel). 2024 Jun 5;14(6):729. doi: 10.3390/life14060729 (PMC11205069; doi:10.3390/life14060729)
Supplement: Supplementary file 1 [file life-14-00729-s001.zip › Table S1.pdf]

Table S1. List of primer sequences.

	Forward primer (5'-3')	Reverse primer (5'-3')
mSirt1	TCGTGGAGACATTTTTAATCAGG	GCTTCATGATGGCAAGTGG
mSirt2	CACTACTTCATCCGCCTGCT	CCAGCGTGTCTATGTTCTGC
mSirt3	GGCTCTATACACAGAACATCGAC	TAGCTGTTACAAAGGTCCCGT
mSirt4	CCAAAGCAGGGAGTCAGC	GCAGTCTGCTCCCCACAG
mSirt5	CCAGCTTTAGCAGGAAAAGG	GACTGGGATTCTGGCGTCT
mSirt6	ACGCGGATAAGGGCAAGT	CTCCCACACCTTGCGTTC
mSirt7	AGAAGCGTTAGTGCTGCCG	GAGCCCGTCACAGTTCTGAG
mG6Pase	TCTGTCCCGGATCTACCTTG	GTAGAATCCAAGCGCGAAAC
mSrebp1c	GATGTGCGAACTGGACACAG	CATAGGGGGCGTCAAACAG
mAcc	TCTCTGGCTTACAGGATGGTTTG	GAGTCTATTTTCTTTCTGTCTCGA CCTT
mTnfα	CCCTCACACTCACAAACCAC	ACAAGGTACAACCCATCGGC
mIl1b	GAAATGCCACCTTTTGACAGTG	TGGATGCTCTCATCAGGACAG
mMmp2	ACGATGATGACCGGAAGTGG	GTGTAGATCGGGGCCATCAG
mβ-actin	AGCCATGTACGTAGCCATCC	GCTGTGGTGGTGAAGCTGTA
G6Pase promoter	GCTGTTTTTGTGTGCCTGTT	TGCTATCAGTCTGTGCCTTG
Intergenic control	ATTTTGTGCTGCATAACCTCCT	TAGCAACATCCTAAGCTGGACA
